# Defining the Baseline and Oxidant Perturbed Lipidomic Profiles of *Daphnia magna*

**DOI:** 10.3390/metabo7010011

**Published:** 2017-03-15

**Authors:** Nadine S. Taylor, Thomas A. White, Mark R. Viant

**Affiliations:** School of Biosciences, University of Birmingham, Edgbaston, Birmingham B15 2TT, UK; n.s.taylor@bham.ac.uk (N.S.T.); tom.andrew.white@gmail.com (T.A.W.)

**Keywords:** *Daphnia magna*, lipidome, nESI, DIMS, oxidation, lipid peroxidation, oxidative stress, copper, in vivo, in vitro

## Abstract

Recent technological advancement has enabled the emergence of lipidomics as an important tool for assessing molecular stress, one which has yet to be assessed fully as an approach in an environmental toxicological context. Here we have applied a high-resolution, non-targeted, nanoelectrospray ionisation (nESI) direct infusion mass spectrometry (DIMS) technique to assess the effects of oxidative stress to *Daphnia magna* both in vitro (air exposure of daphniid extracts) and in vivo (Cu^2+^ exposure). Multivariate and univariate statistical analyses were used to distinguish any perturbations including oxidation to the *D. magna* baseline lipidome. This approach enabled the putative annotation of the baseline lipidome of *D. magna* with 65% of the lipid species discovered previously not reported. In vitro exposure of lipid extracts to air, primarily to test the methodology, revealed a significant perturbation to this baseline lipidome with detectable oxidation of peaks, in most cases attributed to single oxygen addition. Exposure of *D. magna* to Cu^2+^ in vivo also caused a significant perturbation to the lipidome at an environmentally relevant concentration of 20 µg/L. This nESI DIMS approach has successfully identified perturbations and oxidative modifications to the *D. magna* lipidome in a high-throughput manner, highlighting its suitability for environmental lipidomic studies.

## 1. Introduction

Utilisation of a more mechanistically based framework for elucidating the adverse effects of toxicants is encapsulated in the Adverse Outcome Pathway (AOP) concept [1]. AOPs provide a knowledge management tool to support the understanding of how a perturbation to normal biological functions can lead, through a series of key events of increasing biological hierarchy, to an adverse phenotypic outcome [2,3]. The discovery of these key events (KEs), however, remains a significant challenge. In particular, there is considerable interest in discovering molecular KEs in environmental organisms that could serve as indicators of organism health and ultimately contribute to environmental risk assessment. The implementation of such molecular KEs into water quality legislation, such as the European Water Framework Directive [4], could also provide the environmental regulator with informative new diagnostic and prognostic tools. Non-targeted omics technologies—including transcriptomics, proteomics, metabolomics and lipidomics [5,6,7,8]—are ideally suited to discover molecular responses to toxicants and therefore represent an exciting opportunity to address this knowledge gap in molecular KEs.

The field of lipidomics has recently emerged as an important component within the broader omics toolkit [9,10,11]. Advances in electrospray ionisation mass spectrometry (ESI/MS), tandem mass spectrometry (MS/MS) and MS in combination with liquid chromatography (LC-MS) have facilitated the more comprehensive identification and quantification of lipid molecular species in biological systems in a relatively high-throughput manner [12,13,14] Non-targeted lipidomics approaches have primarily been adopted in human health and disease [15], for example in the pathological assessment of diabetes, atherosclerosis and Alzheimer’s disease [6], yet relatively few studies have been related to the environmental sciences [16,17,18]. Despite being an essential dietary component in freshwater food webs, relatively little is known about the lipid composition of *Daphnia* spp. or the perturbations to the *Daphnia* lipidome under environmental stress [19,20,21,22]. This is arguably even more surprising given the importance of *Daphnia* as a keystone species in international ecotoxicology testing [23], its listing by the US National Institutes of Health as a model organism for human health, and because it was the first crustacean to have its genome sequenced [24].

Oxidative stress is a well-known mode of action for several toxicants in which reactive oxygen species (ROS) overwhelm cellular protective mechanisms. An important consequence of ROS production is non-enzymatic lipid peroxidation [25], in which the sn1 and sn2 unsaturated fatty acyl chains attached to the glycerol backbone in phospholipid species are targeted. Hydrogen atoms adjacent to carbon double bonds on the acyl chains are abstracted by ROS to form radical species, and these lipid radicals then propagate, resulting in extensive lipid peroxidation and oxidative damage [26]. This mechanism has been shown to be conserved across in vitro and in vivo studies [27]. Direct measurement of the primary (hydroperoxide) products is difficult due to their labile, highly reactive nature [28,29]. Mass spectrometry based lipidomics offers great potential to provide mechanistic insights into toxicity pathways, for example by detecting and even localising the occurrence of lipid peroxidation during oxidative stress [27] as well as the direct detection of lipid hydroperoxides [30]. Further benefits in analytical sensitivity can be gained by utilising nanoelectrospray (nESI) MS based lipidomics of small samples [31]. While measuring the content and distribution of lipids and their oxidation products has been applied successfully in human health diagnostics, including apoptosis signalling and neuropathology [32,33,34], this approach has rarely been applied in environmental toxicology [19,20,21,35].

Here we report the first application of high resolution, high mass accuracy nESI direct infusion mass spectrometry (DIMS) as a high-throughput approach for directly measuring the lipidome of *Daphnia magna*, with the objective to demonstrate this technology’s value in detecting toxicant induced oxidative stress [36]. We utilise nESI Fourier transform ion cyclotron resonance (FT-ICR) mass spectrometry, which is a proven approach for the analysis of complex mixtures due to its high mass accuracy and resolution [37], aiding the measurement and annotation of complex lipid extracts. This approach has previously been successful in environmental metabolomics, for example to discover the polar metabolite changes in toxicant exposed *D. magna* [38,39,40]. First, we used a shotgun nESI DIMS approach to determine the baseline lipidome of the freshwater model organism, *D. magna*. Next, a simple model was employed to prove the capability of the DIMS technology by detecting the oxidation of biological lipids in vitro; specifically, polar lipids as a representative class of compounds extracted from *D. magna* were oxidised via exposure to a harsh stressor (oxygen in air) and the resulting chemical mixture was characterised. This in vitro study was conducted primarily as a methodological test of the oxidative lipidomics workflow, not to reveal novel biology. Lastly, we sought to induce and observe a perturbation of the *D. magna* lipidome in vivo using toxicologically relevant concentrations of copper (Cu) as a model environmental toxicant. This in vivo study was conducted primarily as a real-world application of the oxidative lipidomics workflow. Cu is known to cause oxidative stress via its participation in redox cycling and the formation of ROS [41], evidence for which has been reported previously in the polar metabolome of *D. magna* [38].

## 2. Results

### 2.1. Characterisation of the Daphnia magna Baseline Lipidome

**Figure 1:**
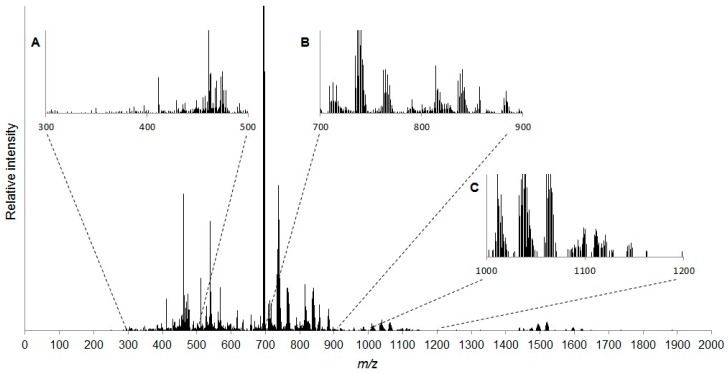
Representative mass spectrum of *D. magna* lipid extract. Spectra were collected in *m*/*z* range 70–2000. Insets show zoomed regions containing peak clusters (**A**) *m*/*z* 300–500; (**B**) *m*/*z* 700–900; and (**C**) *m*/*z* 1000–1200. Putative lipid annotations are listed in Supplementary Information; Table S2.

Following data processing of 10 replicate samples (including replicate, sample and blank filtering steps) and internal mass calibration (see Supplementary Information; Table S1), 1638 spectral peaks were retained to form the baseline lipidome of *D. magna* (Figure 1). Putative annotation of spectral peaks by matching to the LipidMAPS database resulted in 32% (532 peaks) of the *m*/*z* values being assigned at least one lipid name (see Supplementary Information; Table S2). Our putative annotation workflow discovered at least a single adduct of 374 individual polar lipids in the baseline lipidome of *D. magna* with all of the major phospholipid classes represented (Table 1).

**Figure 2:**
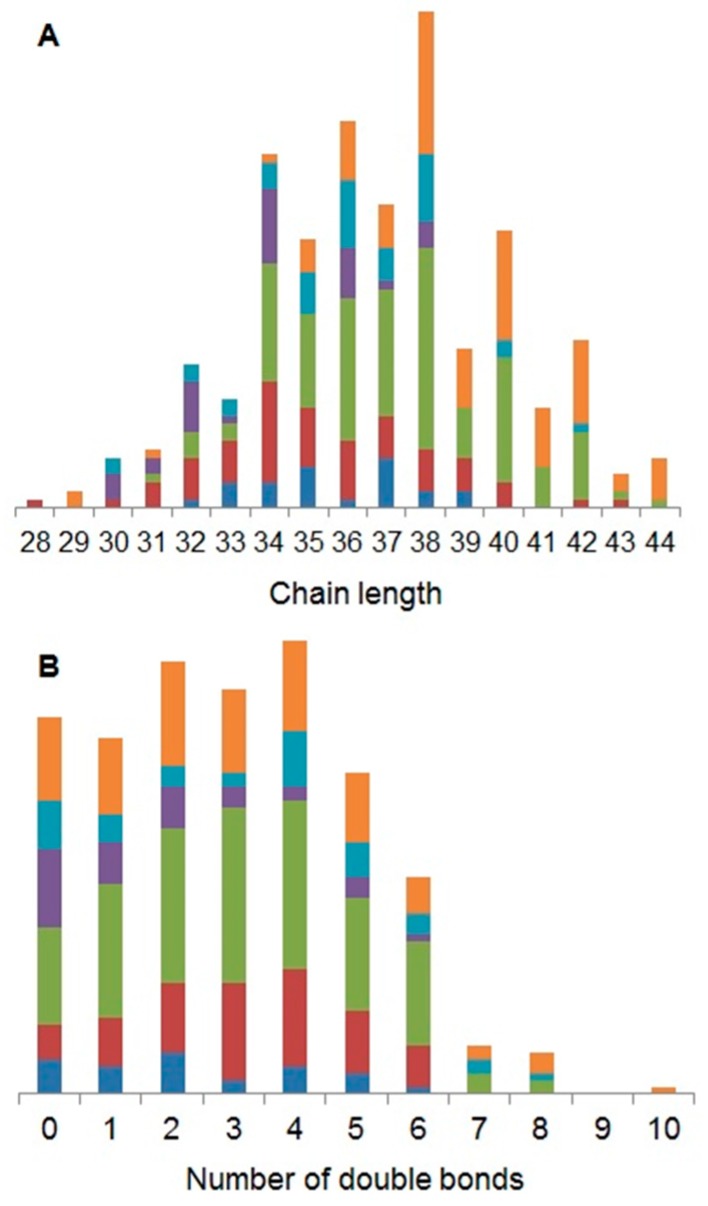
Bar charts showing the characteristics of the putatively annotated phospholipids in the *D. magna* baseline lipidome, specifically the distributions of chain lengths (**A**) and number of double bonds (**B**). Phospholipid class is shown by colour and order, bottom to top: PA (blue), PC (red), PE (green), PG (purple), PI (turquoise) and PS (orange).

Based upon the putative annotations of lipids in this study, it is evident that the most abundant carbon chain lengths are 34 in PG (phosphatidylglycerol), 36 in PC (phosphatidylcholine) and PE (phosphatidylethanolamine), 38 in PI (phosphatidylinositide), 40 in PS (phosphatidylserine) and 37 in PA (phosphatidic acid) species (Figure 2A). These chain lengths are expected to widely occur, as they are all potential products of even integers between 16 and 20, the major radyl chain lengths in *D. magna* [22,42]. Saturation was split similarly across lipid classes with the following dominant double bond annotations: two in PA, PG and PS; three in PC; and four in PE and PI. A total intensity comparison of double bond annotation creates a bell shaped curve with the abundance order 4 > 3 > 5 > 2 > 6 > 1 > 0 > 7 > 8 > 10 with no annotations of nine double bonds (Figure 2B). Again, our observations are consistent with previously reported saturations of total fatty acids (FA) [22,42].

### 2.2. In Vitro Oxidation of the Daphnia magna Lipidome

**Figure 3:**
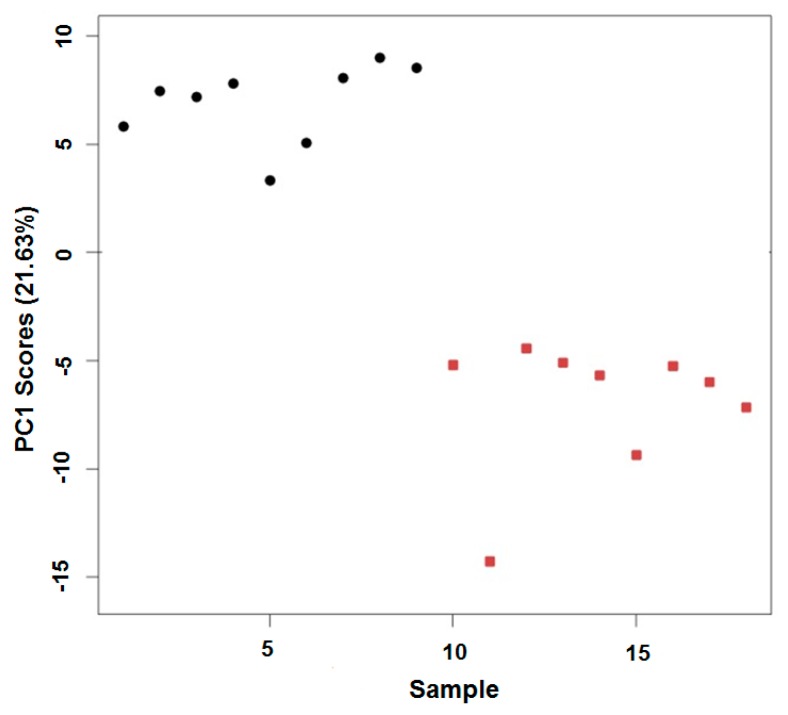
PCA (principal component analysis) scores plot showing sample identity vs. principal component 1 (PC1) score from the in vitro air oxidation of *D. magna* lipid extracts. Control samples (1–9) are denoted by black circles (●); exposed samples (10–18) are denoted by red squares (■). Data are presented in this format as no separation of groups was visible in the higher order PCs.

Following the nESI FT-ICR-MS analysis of the control or 168-h air-exposed *D. magna* lipid samples, an intensity matrix comprising 1097 peaks was generated (see Supplementary Information; Table S3). Prior to statistical analysis to attempt to discover the effects of treatment, an assessment of each sample for missing intensity values resulted in the removal of a single sample from each treatment group to maintain high spectral quality (i.e., few missing values); therefore, subsequent statistical analyses were conducted on *n* = 9 biological replicates for both the control and the air-exposed groups. Principal component analysis (PCA) was then conducted on the glog transformed dataset to visualise any differences between the control and oxidatively-modified lipid extracts (Figure 3). Highly significant separation was observed between these two groups along PC1 (*p* = 3.86 × 10^−9^), revealing that the baseline lipidome of *D. magna* was significantly perturbed in vitro following oxidation through air exposure.

Next, univariate statistical analysis (Student’s *t*-test) was used to identify which peaks showed significant intensity changes between the control and air-exposed groups. Intensity fold-changes and false-discovery rate (FDR) corrected *p*-values (<0.05) were calculated for each peak (see supplementary information; Table S4). Considering all of the spectral peaks detected, 479 peaks (44% of the dataset) were found to change significantly following exposure to air with predictably large associated fold changes (log_2_(fold changes); min: −13.4, max: 15.3). The binary logarithm (log_2_) was used as a convenient way to compare mass spectral intensity changes between exposure and control groups:

$$\log_{2}\mathrm{FC}\left(\mathrm{fold}\;\mathrm{change}\right)=\log_{2}\left(\frac{I_{C}}{I_{E}}\right).$$

A twofold increase in the intensity of a mass spectral feature in the exposure group (*I*_E_) relative to the intensity of the same feature in the control group (*I*_C_) would yield a log_2_FC of +1; a twofold decrease yields a log_2_FC of −1; and no change yields a log_2_FC of 0. The major change in lipid extract composition reflects the effectiveness of in vitro oxidative stress. Additionally, some spectral peaks were only detected in the control or air-exposed groups (118 peaks). Peaks only present in the exposure group could be oxidised lipids, which should not be present in the control sample. Peaks only present in the control group could be those that were so heavily oxidised that the parent compound fell below the limit of detection.

**Figure 4:**
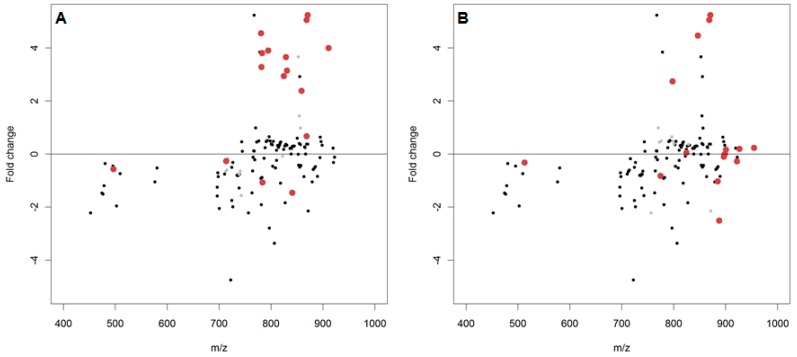
The log_2_(fold changes) of putatively annotated polar lipids and the potentially oxidised products thereof from *D. magna* lipid extracts. Red dots denote a spectral peak separated from the putatively annotated peak by either (**A**) a single oxygen (*m*/*z* 15.99491) or (**B**) a double oxygen (*m*/*z* 31.98983). Isomeric overlap between an annotated species and oxidised product is denoted by a light grey dot.

In order to apply a more constrained lipid oxidation search, the baseline lipidome for *D. magna* (see Supplementary Information; Table S5) was used as an unmodified lipid list and single and double oxygen additions were identified to indicate potential oxidised products. In the air-exposed *D. magna* lipid extracts (Figure 4), 26 oxidised compounds were putatively observed of which 13 had log_2_FC > 1 (corresponding to a greater than twofold positive change) and only two of which were annotated, in contrast to 13 with log_2_FC < 1 of which eight were annotated. However, the 25 double oxygen additions (i.e., lipid peroxidation) did not follow the same trend with only four log_2_FC > 1, although none were annotated, and 21 log_2_FC < 1, with nine putatively annotated.

### 2.3. In Vivo Oxidation of the Daphnia magna Lipidome by Copper

**Figure 5:**
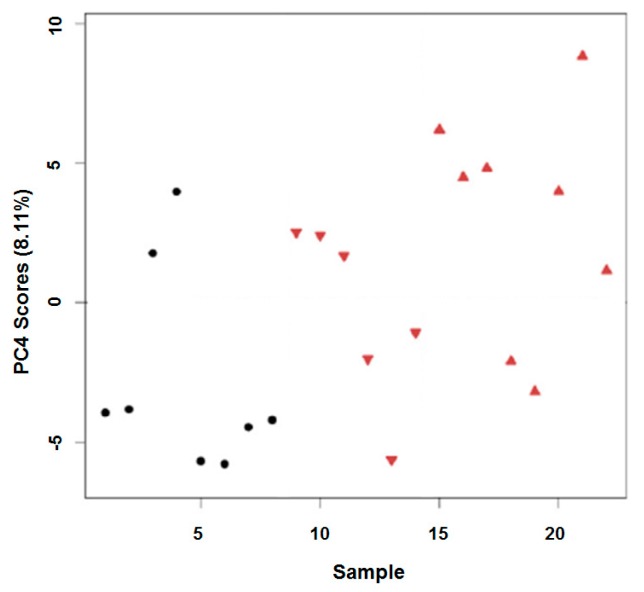
Scores plot showing sample vs. principal component 4 (PC4) for in vivo *D. magna* exposures to CuSO_4_. Control samples (1–8) are denoted by black circles (●), CuSO_4_ exposed samples (8–21) are denoted by red triangles (2 µg/L ▼, 20 µg/L ▲).

Dried lipid extracts of *D. magna* exposed to 0, 2 or 20 µg/L CuSO_4_ were analysed by nESI FTICR-MS and processed intensity matrices were assessed for missing values. Any samples of poor spectral quality were removed from subsequent analyses, resulting in a final data matrix consisting of *n* = 8 (control; 0 µg/L CuSO_4_), *n* = 6 (2 µg/L CuSO_4_) and *n* = 8 (20 µg/L CuSO_4_) samples (see Supplementary Information; Table S6). PCA was conducted to observe the differences between control and copper exposed groups. Significant treatment effects were discovered along PC4 (*p* = 0.0189; Figure 5), with Tukey’s post hoc analysis confirming the separation was between control and high-dosed groups. However, there was considerably less perturbation to the lipidomic profile of *D. magna* in response to in vivo oxidative stress (from copper) compared to in vitro oxidation (via air) with only 11 of 1289 peaks changing significantly following exposure to CuSO_4_ (see supplementary information; Table S7). Correspondingly, very few of the peak intensity changes between control and high dose CuSO_4_ (log_2_FC) were above 1 or below −1 (min: −5.1; max: 3.0).

**Figure 6:**
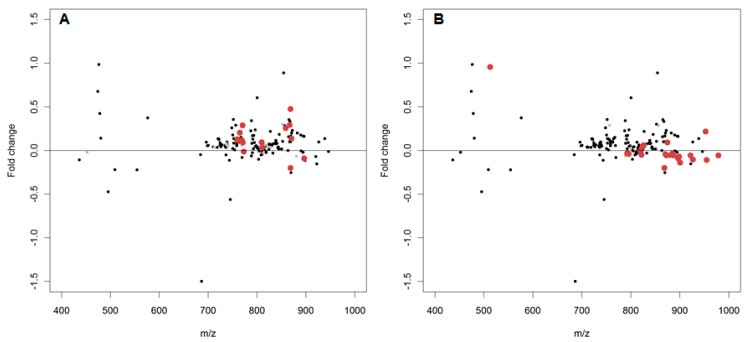
The log_2_(fold changes) of putatively annotated polar lipids and the potentially oxidised products thereof from *D. magna* lipid extracts. Red dots denote a spectral peak separated from the putatively annotated peak by either (**A**) a single oxygen (*m/z* 15.99491) or (**B**) a double oxygen (*m/z* 31.98983). Isomeric overlap between an annotated species and oxidised product is denoted by a light grey dot.

Targeted annotation of oxidatively modified peaks also yielded little of note. Over 100 spectral peaks were matched to the baseline lipidome of *D. magna* with many single and double oxygen additions putatively annotated from these matched peaks. However, there was no identifiable trend in the log_2_FC, with no spectral peaks changing positively or negatively by as much as twofold (Figure 6).

## 3. Discussion

### 3.1. Characterisation of the Daphnia magna Baseline Lipidome

While the fatty acid (FA) composition of *Daphnia* is known to be largely determined by the FA profile of their food source [22,42,43], the phospholipid origins of these FA moieties have also been identified. There are five major phospholipid species commonly found in *D. magna*: PE, PC, PS, PI and PG [22]. Further studies of the lipid composition of *D. magna* discovered that of these five classes, PEs and PCs make up ca. 90% of all phospholipids [19]; several other lipid classes have also been determined in *D. magna* including PAs, lysophosphatidylcholine (LPC), lysophosphatidylethanolamine (LPE) and sphingomyelin (SM) [20,21,35]. Of the nine lipid classes previously reported to be present in *D. magna*, we have measured eight of them, with SM being the only class not detected by our methods. In total, 65% of the individual lipid species putatively annotated in the current study have not been previously reported [21].

### 3.2. In Vitro Oxidation of the Daphnia magna Lipidome

Mass spectrometry can be used to detect oxidised products of phospholipids which occur from lipid peroxidation, with double oxygen addition the most abundant product followed by reduction to single oxygen addition [26]. Therefore, we sought to find potentially oxidised lipids by relating peaks with *m*/*z* differences of one or two oxygen atoms (15.99491 or 31.98983 Da, <2 ppm error) within the peak matrix. Numerous putative single and double oxygen mass differences were observed. Assuming that if the control samples contain only unoxidised lipids and the air-exposed samples have many oxidatively modified lipids, then unoxidised lipids should have high negative log_2_(fold changes) and oxidatively modified lipids have high positive fold changes. In fact, this was not always the case as the unmodified and oxidised annotated lipids appeared with both positive and negative fold changes. In addition, there were some peaks that were putatively annotated as oxidation products of lower *m*/*z* peaks but were themselves related to even higher *m*/*z* peaks, which could be oxidised products that were oxidised further. Inherent single and double oxygen structural differences between different lipids would also be expected to occur, adding complexity (and an elevated false positive error rate) to this analysis; for example, diacyl phospholipid species will always be observed at a single oxygen *m*/*z* difference above the corresponding plasmalogen and ester linked phospholipid species. The species with positive log_2_FC from the control to exposure group were predominantly single oxygen additions, indicating that the anticipated products of lipid peroxidation, namely lipid peroxides (+2O), subsequently formed lipid hydroxides via reduction reactions [44].

### 3.3. In Vivo Oxidation of the Daphnia magna Lipidome by Copper

Phospholipids are present in all organisms and are the major constituent of cell membranes [45], thus the effects of toxicants to the polar lipidome of *D. magna* are integral to understanding lipid peroxidation, yet only a few such studies have been reported [19,20,21,35]. More broadly, the application of metabolomics to discover evidence of oxidative stress and membrane insult in fish liver [46] and gill [47] have been reported, yet no induction of lipid peroxidation was observed. In this study, *D. magna* were exposed to Cu at toxicologically relevant concentrations, supported by previously reported studies [38,48,49]. Specifically, the exposure concentrations used here were known to be sub-lethal to *D. magna* when considering standard OECD (Organisation for Economic Co-operation and Development) test endpoints; however, the highest concentration (20 µg/L Cu^2+^) has been shown to cause significant perturbation to the metabolome of *D. magna* [38]. The significant separation of the high dose group from the control group indicates that exposure to Cu causes a similar perturbation to the *D. magna* lipidome. The relative lack of significantly changing lipid species in vivo (compared to in vitro) could arise for a number of reasons, including the shorter duration of the acute toxicity exposure (to match OECD recommended guidelines for *Daphnia* acute toxicity testing [50]) or indeed because of the capability of a living organism to respond defensively to oxidative stress.

Lipid peroxidation was not observed in any significant way with fold changes largely less than twofold positively or negatively. As previous studies have reported oxidative stress and resulting lipid peroxidation as toxicological effects of copper exposure using non-lipidomic methodologies [51,52], it is possible that lipid peroxides are present but not observed in this study. Therefore, use of these DIMS techniques for analysis of phospholipid peroxides and related compounds in vivo may be undermined by the low abundance of these particular molecular species [33].

## 4. Materials and Methods

### 4.1. Daphnia Culturing and Lipid Extraction

Cultures of *D. magna* were maintained as previously reported [38]. For the annotation of the *D. magna* baseline lipidome and for all of the exposure experiments described below, neonates (3rd brood, <24 h old) were harvested in groups of 30, flash frozen in liquid nitrogen and stored at −80 °C prior to extraction. A methanol:chloroform:water biphasic extraction method was used to obtain hydrophobic (lipid) and hydrophilic fractions from frozen tissue as described previously [38,53]. As only the lipid fraction was required for this study, the lower predominantly chloroform layer (200 μL) that contained the hydrophobic compounds was removed to a glass vial, avoiding disturbance of the protein layer. The chloroform was removed under anoxic conditions in a nitrogen blow-down sample concentrator (Techne, Bibby Scientific, Stone, UK) to minimise oxidation artefacts. All dried samples were used immediately or stored at −80 °C for a maximum of one month.

### 4.2. In Vitro Oxidation of Daphnia Lipid Extracts

The dried *Daphnia* lipid extracts (*n* = 10) were retrieved from −80 °C storage, the lids were removed from the vials to expose lipid residues to air [54] and left lightly covered on the laboratory benchtop at room temperature for 168 h. Air exposed lipid extracts were then resuspended, analysed, processed and annotated as described below, alongside control lipid extracts (*n* = 10), which were retrieved from −80 °C storage immediately prior to analysis.

### 4.3. In Vivo Oxidation of the Daphnia lipidome

For the in vivo oxidation of *D. magna*, groups of 30 neonates (<24 h, *n* = 8) were exposed to copper sulfate (CuSO_4_; calculated as Cu^2+^ ions) for 48 h. Preliminary toxicity studies determined a no observed adverse effect level (NOAEL) for CuSO_4_ at 20 µg/L [38]. Based on these data, two exposure concentrations were used in this experiment, the NOAEL (20 µg/L) and 1/10 NOAEL (2 µg/L). After 48 h neonates were harvested, lipids extracted and samples analysed as detailed above.

### 4.4. Direct Infusion FT-ICR Mass Spectrometry

Dried lipid residues were dissolved on ice in re-suspension solution for negative ion analysis (MeOH:CHCl_3_, 2:1 with 5 mM ammonium acetate; 50 μL). Samples were vortexed and centrifuged (10 min, 4000 rpm, 4 °C) to remove solid contaminants. A 384-well polymerase chain reaction (PCR) plate (ABgene, Thermo Fisher Scientific, Waltham, MA, USA) was placed on ice (10 min) before sample aliquots (6 μL) were transferred by manual pipette in quadruplicate to individual wells with carbon pipette tips (Advion, Harlow, UK) and immediately covered with strips of self-adhesive foil. Following completion of sample transfer, the self-adhesive strips were replaced by a single heat-sealed foil sheet (ALPS 50V, Thermo Fisher Scientific, USA). Samples were delivered to a hybrid 7-tesla linear ion trap FT-ICR mass spectrometer (LTQ FT, Thermo Scientific, Bremen, Germany) with no prior separation via a nESI source (Advion, Harlow, UK) in negative ion mode. FT-ICR MS analysis was conducted in such a way to allow data processing by the previously reported selective ion monitoring (SIM)-stitch methodology, optimised for analysis of polar fractions [37,55,56]. Mass spectra were collected as transients across the available range (70–2000 *m*/*z*) in ten, “wide-SIM” windows overlapping by 30 Da. Preliminary lipidomic mass spectra of *D. magna* in negative ion mode show peaks predominantly between 600 and 900 *m*/*z* and, as such, windows in this region were 100 *m*/*z* wide, reported as optimal for this instrumental set up [37]. Windows outside this range were widened to avoid under fill, a scenario where too few ions are collected during the acquisition time leading to unreliable peak intensities (*m*/*z*: 70–300, 270–500, 470–670, 640–730, 700–800, 770–870, 840–940, 910–1010, 990–1410, 1380–2000).

### 4.5. Data Processing and Statistical Analysis

An intensity matrix with rows and columns, corresponding to samples and *m*/*z* values of spectral peaks, respectively, was produced along the SIM-stitch workflow [56]. Briefly, transients were averaged, transformed into spectra with Fast Fourier Transform (FFT) [57] and calibrated using a list of defined spectral peaks (see supplementary information) for each SIM window. Calibrated SIM windows were “stitched” together via alignment of peaks in overlapping (30 Da) windows to yield a single spectrum for each replicate of peaks above a selected signal-to-noise ratio (SNR = 10) with regularly observed “high noise regions” removed (*m*/*z* = 74.05–74.2; 90.50–90.58, 101.32–101.42; 101.6–102.1; 105.1–105.5; and 116.37–116.5). Three spectra per sample were selected for further processing, allowing removal of low quality spectra, based on spray stability, total ion current (TIC) profile shape and file size. Spectral reliability was enhanced using a three-step filtering process. A replicate filter combined triplicate spectra, retaining only peaks present at least two of the three of the spectra within a specified error range (<2 ppm) yielding a single robust spectrum for individual sample. A blank filter then removed peaks with intensities less than 10-fold above that in the extract blank as these were considered to be artefacts of the extraction procedure. Finally a sample filter removed those peaks not present in 85% within a single group.

The intensity matrix produced requires further processing to allow statistical analyses. Probable quotient normalisation (PQN) used intensities of ubiquitous peaks to remove variation arising from unavoidable sample concentration differences [58]. Quotients of intensity over mean intensity across all spectra were calculated for each peak present in 100% of samples, and the median of all quotients within a SIM-window was defined as the most probable quotient and used to normalise all peaks within that window. Missing intensity values occurred in the data matrix when no peak of sufficient intensity and reproducibility was detected at the specified *m*/*z* for a particular sample. To avoid exaggerated fold changes, these missing values were replaced with the minimum intensity from the matrix, representing a minimum detection level. Finally, multivariate statistics can be dominated by more intense peaks with intrinsically higher variation; to counter this and reduce technical variation, the dataset underwent generalised logarithm (glog) transformation [59]. Glog involves a transformation parameter (λ), calibrated on a subset of data containing only technical variation, being applied to the whole dataset, emphasising biological variation in subsequent multivariate statistical analyses. Univariate statistics were applied to pre-glog lipidomic datasets, Student’s *t*-test or ANOVA were used to identify statistically significant fold changes from control to treated in single- and multi-dose experiments respectively. Due to the high number of fold changes being assessed, large FDR of significance require control; as such, *p*-values were modified using a sequential Bonferroni-type procedure reported previously [60]. PCA was used as an unbiased method to highlight the peaks most discriminating between treatment groups. All PCA models within this study contain six principal components and were created by the PLS toolbox (version 8.0.2, Eigenvector Research, Manson, WA, USA) in the MATLAB environment (version 8.3 R2014a, MathWorks, Natick, MA, USA). ANOVA or *t*-test of mean intragroup PC score was used to assess significance of group separation

### 4.6. Determining the Baseline Lipidome of D. magna

Putative peak annotation (Metabolomics Standards Initiative; MSI level 2 [61]) used the accurate *m*/*z* values arising from the DIMS analysis to create a peak list with associated mean intensities, which was then input into MI-Pack software version 2 beta [62]. This software has previously been used in conjunction with the Kyoto Encyclopedia of Genes and Genomes (KEGG) LIGAND database to putatively annotate polar metabolites in *D. magna* [38]. MI-Pack (version 2 beta) incorporates the database from the lipid metabolites and pathways strategy (LipidMAPS) allowing increased annotation of lipid metabolite extracts [63]. In brief, MI-Pack calculated accurate *m*/*z* values for probable negative ions ([M − H]^−^, [M + ^35^Cl]^−^, [M + ^37^Cl]^−^, [M + Acetate(Ac)]^−^, [M + K-2H]^−^, [M + Na-2H]^−^) of all compounds in the KEGG LIGAND and LipidMAPS databases and assigned these to any peaks within a set ppm error range (<2 ppm).

For the identification of the baseline lipidome of *D. magna*, accurate *m*/*z* data and MS/MS scans were recorded in negative ion mode for each SIM-window used in the lipidomic MS analysis. Accurate *m*/*z* values were matched against the entire LipidMAPS and KEGG databases via MI-Pack to give putative peak annotations. Fragmentation patterns were then used to confirm or refute these annotations. Deprotonated fatty acids were confirmed by the loss of CO_2_ and H_2_O and the loss of propanoate (M − (CH_2_)_2_CO_2_), which has been reported previously [64]. Phospholipids were identified by appropriate carboxylate ions and corresponding neutral losses which matched the sn1 and sn2 chains in the putative ID. Additionally, loss of serine (M − 87 Da) was indicative of [PS − H]^−^, loss of methyl acetate (M − 74 Da) of [PC + Ac]^−^, and loss of inositol (M − 163 Da) of [PI − H]^−^. LipidBlast was also used to match in silico fragmentation patterns to experimental fragmentation patterns using a precursor ion accuracy cut off of <2 ppm. A further MS/MS analysis was implemented to separate the isomeric species [PS − H]^−^ and [PC + Ac]^−^. Spectral peaks were isolated in 100-Da wide SIM windows and fragmented with CID (collision energy = 80 kV). Fragments were collected in the FT-ICR MS in windows 100-Da wide and 80-Da lower than the isolation window. The resulting fragmentation spectra were processed analogously to DIMS spectra along the SIM-stitch workflow. The resulting mass list containing the major fragments from all spectral peaks was transformed with the exact mass of methyl acetate (*m*/*z* 74.03677) and serine minus H_2_O (*m*/*z* 87.03203) to yield two new peak lists containing potential [PC + Ac]^−^ and [PS − H]^−^ ionic masses respectively. These peak lists were matched against LipidMAPS phospholipid database using MI-Pack with a mass error cut-off <2 ppm. The “PC” and “PS” peak lists were used to confirm or refute putative PC and PS annotations from the baseline lipidome annotation.

### 4.7. Putative Annotation of Potential Oxidation Products

Spectral peaks which appeared at a mass difference of one or two oxygen atoms (+O = 15.99492 *m*/*z*, +2O = 31.98983 *m*/*z*) higher than a second peak within a mass error range of 2 ppm were annotated by a bespoke script in MATLAB and highlighted as potentially oxidised products. Spectral peaks which appeared at +O or +2O above a second peak and −O or −2O below a third peak were also highlighted. These spectral peaks could be oxidatively modified lipids that were subsequently oxidised a second time or lipids with inherent oxygen differences that were oxidised. Targeted annotation of oxidised lipids was achieved by a similar method by assigning +O and +2O to spectral peaks which were appropriate mass differences higher than lipids annotated in the baseline lipidome. Annotated lipids that appeared at +O and +2O mass differences above a second annotated lipid were also highlighted, and these would likely mask oxidation of the second lipid, as oxidised products are unlikely to be more abundant than an endogenous species.

## 5. Conclusions

The current study has successfully used nESI DIMS to analyse small tissue extracts (equivalent of 225 µg dry mass and 1125 µg wet mass) in a less than 3 min analysis, highlighting the suitability of this analytical approach for high-throughput profiling of the *Daphnia* lipidome. We have identified ion forms of all of the major phospholipid classes in *D. magna* with chain parity, length and saturation agreeing with previously reported total fatty acid and phospholipid classes, with even chains dominating and a majority of the lipids exhibiting some unsaturation [42]. While the bulk fragmentation methodology added confidence to the PS and PC annotations in the *D. magna* baseline lipidome, the comprehensive identification of lipid species (i.e., to MSI level 1 [61]) would require separation and MS/MS fragmentation techniques as well as comparison of the spectra to those obtained from pure standards, if those chemicals are commercially available.

Perturbation of the *D. magna* baseline lipidome (of which 44% of the detectable peaks significantly changed) was induced via exposure of the lipid extracts to air. Statistical analysis of the data did not reveal oxidative modifications that changed significantly despite many hundreds of spectral peaks and large significant fold changes between control and exposed groups. A more targeted inspection of the baseline lipidome, however, showed large positive fold changes of putatively annotated oxygen additions between control *D. magna* lipid extracts and air exposed extracts. The lipid species with positive log_2_FC from the control to exposure group were predominantly single oxygen additions. This indicates that, while lipid peroxidation should initially lead to lipid peroxides (+2O) as the primary product, lipid hydroxides (+O) are subsequently formed via reduction reactions [44]. Direct treatment of *Daphnia* by copper induced relatively minor changes in the lipidome at the exposure concentrations used.

## Figures and Tables

**Table 1:** Summary of putatively annotated polar lipids in the baseline analysis of *D. magna*. Annotations include lyso- and plasmalogen species. Lipid annotations are counted only once regardless of the multiple ion forms (adducts, isotopes) that were detected in the mass spectra. Phosphatidic acid (PA), phosphatidylethanolamine (PE), phosphatidylcholine (PC), phosphatidylglycerol (PG), phosphatidylinositide (PI), phosphatidylserine (PS), monogalactosyldiacylglycerol (MGDG).

Lipid Class	PA	PC	PE	PG	PI	PS	MGDG	Total
Number of putatively annotated lipid species	30	87	107	32	35	80	3	374

## Supplementary Materials

The following are available online at www.mdpi.com/2218-1989/7/1/11/s1: Table S1: Identified lipid species used as internal calibrants for *D. magna* lipidome data processing, Table S2: Putative identification of lipids detected in the mass spectra of *D. magna*, Table S3: Putative identification of lipids detected in the mass spectra of *D. magna* lipid extracts exposed to air in vitro, Table S4: Summary of the top 20 (ranked according to the greatest fold change) putatively identified lipids of *D. magna* exposed to air in vitro, Table S5: Annotated baseline lipidome of *D. magna*, Table S6: Putative identification of lipids detected in the mass spectra of *D. magna* lipid extracts exposed to CuSO_4_ in vivo, Table S7: Summary of the top 20 (with the greatest fold change) putatively identified lipids of *D. magna* exposed to CuSO_4_ in vivo.
