# Supplementary material for: Defining the Baseline and Oxidant Perturbed Lipidomic Profiles of Daphnia magna
Source: Metabolites. 2017 Mar 15;7(1):11. doi: 10.3390/metabo7010011 (PMC5372214; doi:10.3390/metabo7010011)
Supplement: Supplementary file 1 [file metabolites-07-00011-s001.zip › Table S4.pdf]

Table S4. Summary of the top 20 (ranked according to the greatest fold change) putatively identified lipids of *D. magna* exposed to air *in vitro.*

Observed		Statistics		Annotation				
**mz**	**Average intensity[a]**	**Fold change[b]**	**p-value[c]**	**Empirical formula**	**Ion form**	**Theoretical mass (Da)[d]**	**Mass error (ppm)[e]**	**Putative ID**
415.32123	1.53E+05	122.5	0.000	C25H40O	[M+Hac-H]-	415.32177	-1.3	['(-)-Ceriferol 1', '26,27-bisnor-22-dehydro-cholesterol']
415.32123	1.53E+05	122.5	0.000	C27H44O3	[M-H]-	415.32177	-1.3	Too many to list explicitly (see Table S3)
766.50331	9.86E+04	78.9	0.000	C42H74NO9P	[M-H]-	766.50285	0.61	['PS(O-16:0/20:5(5Z,8Z,11Z,14Z,17Z))', 'PS(P-16:0/20:4(5Z,8Z,11Z,14Z))', 'PS(P-18:0/18:4(6Z,9Z,12Z,15Z))']
335.18703	8.23E+04	66.0	0.002	C17H24O3	[M+Hac-H]-	335.18640	1.88	['Sugeonyl acetate']
764.48728	8.05E+04	64.5	0.000	C42H72NO9P	[M-H]-	764.48720	0.11	['PS(P-16:0/20:5(5Z,8Z,11Z,14Z,17Z))']
664.41983	6.90E+04	55.2	0.000	C33H64NO10P	[M-H]-	664.41951	0.48	[' PC(16:0/9:0(COOH))', 'PS(12:0/15:0)', 'PS(13:0/14:0)', 'PS(14:0/13:0)', 'PS(15:0/12:0)']
664.41983	6.90E+04	55.2	0.000	C31H60NO8P	[M+Hac-H]-	664.41951	0.48	['PE(12:0/14:1(9Z))', 'PE(14:1(9Z)/12:0)']
381.26457	5.66E+04	45.3	0.000	C22H38O5	[M-H]-	381.26465	-0.21	['14-F2-dihomo-IsoP', '16,16-dimethyl-PGE1', '16,16-dimethyl-PGF2beta', '17-F2-dihomo-IsoP', '1a,1b-dihomo-PGE1', '1a,1b-dihomo-PGF2alpha', '20-ethyl PGF2alpha', '7-F2-dihomo-IsoP', 'Carboprost methyl (USAN)', 'Unoprostone (INN)']
381.26457	5.66E+04	45.3	0.000	C20H34O3	[M+Hac-H]-	381.26465	-0.21	['(+)-Villanovane-13alpha,19-diol', '(-)-2,7-Dolabelladiene-6beta,10alpha,18-triol', '(-)-Spongiane-15,16-diol', '12R-HETrE', '15(S)-Hydroxyeicosatrienoic acid', '5S-HETrE', '8S-HETrE']
768.51926	5.60E+04	44.8	0.000	C42H76NO9P	[M-H]-	768.51850	0.99	['PS(O-16:0/20:4(5Z,8Z,11Z,14Z))', 'PS(O-18:0/18:4(6Z,9Z,12Z,15Z))', 'PS(P-16:0/20:3(8Z,11Z,14Z))', 'PS(P-18:0/18:3(6Z,9Z,12Z))', 'PS(P-18:0/18:3(9Z,12Z,15Z))']
433.33177	4.98E+04	39.9	0.000	C27H46O4	[M-H]-	433.33233	-1.3	Too many to list explicitly (see Table S3)
433.33177	4.98E+04	39.9	0.000	C25H42O2	[M+Hac-H]-	433.33233	-1.3	['Dolichoic acid-[18-20]']

870.54950	4.72E+04	37.7	0.000	C44H78NO10P	[M+Hac-H]-	870.55019	-0.79	['PS(16:0/22:4(7Z,10Z,13Z,16Z))', 'PS(18:0/20:4(5Z,8Z,11Z,14Z))', 'PS(18:1(9Z)/20:3(8Z,11Z,14Z))', 'PS(18:2(9Z,12Z)/20:2(11Z,14Z))', 'PS(18:3(6Z,9Z,12Z)/20:1(11Z))', 'PS(18:3(9Z,12Z,15Z)/20:1(11Z))', 'PS(18:4(6Z,9Z,12Z,15Z)/20:0)', 'PS(20:0/18:4(6Z,9Z,12Z,15Z))', 'PS(20:1(11Z)/18:3(6Z,9Z,12Z))', 'PS(20:1(11Z)/18:3(9Z,12Z,15Z))', 'PS(20:2(11Z,14Z)/18:2(9Z,12Z))', 'PS(20:3(8Z,11Z,14Z)/18:1(9Z))', 'PS(20:4(5Z,8Z,11Z,14Z)/18:0)', 'PS(22:4(7Z,10Z,13Z,16Z)/16:0)']
767.50647	4.69E+04	37.5	0.000	C37H73O10P	[M+Hac-H]-	767.50799	-1.98	['PG(12:0/19:0)', 'PG(13:0/18:0)', 'PG(14:0/17:0)', 'PG(15:0/16:0)', 'PG(16:0/15:0)', 'PG(17:0/14:0)', 'PG(18:0/13:0)', 'PG(19:0/12:0)']
767.50647	4.69E+04	37.5	0.000	C39H77O12P	[M-H]-	767.50799	-1.98	['PI(O-16:0/14:0)', 'PI(O-18:0/12:0)']
868.53355	4.16E+04	33.3	0.000	C44H76NO10P	[M+Hac-H]-	868.53454	-1.14	['PS(16:1(9Z)/22:4(7Z,10Z,13Z,16Z))', 'PS(18:0/20:5(5Z,8Z,11Z,14Z,17Z))', 'PS(18:1(9Z)/20:4(5Z,8Z,11Z,14Z))', 'PS(18:2(9Z,12Z)/20:3(8Z,11Z,14Z))', 'PS(18:3(6Z,9Z,12Z)/20:2(11Z,14Z))', 'PS(18:3(9Z,12Z,15Z)/20:2(11Z,14Z))', 'PS(18:4(6Z,9Z,12Z,15Z)/20:1(11Z))', 'PS(20:1(11Z)/18:4(6Z,9Z,12Z,15Z))', 'PS(20:2(11Z,14Z)/18:3(6Z,9Z,12Z))', 'PS(20:2(11Z,14Z)/18:3(9Z,12Z,15Z))', 'PS(20:3(8Z,11Z,14Z)/18:2(9Z,12Z))', 'PS(20:4(5Z,8Z,11Z,14Z)/18:1(9Z))', 'PS(20:5(5Z,8Z,11Z,14Z,17Z)/18:0)', 'PS(22:4(7Z,10Z,13Z,16Z)/16:1(9Z))']
580.28909	3.57E+04	28.6	0.004	C24H44NO9P	[M+Hac-H]-	580.28923	-0.23	['PS(18:2(9Z,12Z)/0:0)']
780.51851	2.94E+04	23.6	0.000	C41H72NO7P	[M+Hac-H]-	780.51850	0.02	['PE(P-16:0/20:5(5Z,8Z,11Z,14Z,17Z))']
499.30616	2.81E+04	22.6	0.000	C30H44O6	[M-H]-	499.30651	-0.71	['Hippuristanolide']
846.54966	2.76E+04	22.2	0.006	C42H78NO10P	[M+Hac-H]-	846.55019	-0.63	['PS(14:0/22:2(13Z,16Z))', 'PS(14:1(9Z)/22:1(11Z))', 'PS(16:0/20:2(11Z,14Z))', 'PS(16:1(9Z)/20:1(11Z))', 'PS(17:1(9Z)/19:1(9Z))', 'PS(17:2(9Z,12Z)/19:0)', 'PS(18:0/18:2(9Z,12Z))', 'PS(18:1(9Z)/18:1(9Z))', 'PS(18:2(9Z,12Z)/18:0)', 'PS(19:0/17:2(9Z,12Z))', 'PS(19:1(9Z)/17:1(9Z))', 'PS(20:1(11Z)/16:1(9Z))', 'PS(20:2(11Z,14Z)/16:0)', 'PS(22:1(11Z)/14:1(9Z))', 'PS(22:2(13Z,16Z)/14:0)']
694.44617	2.15E+04	17.2	0.000	C38H66NO8P	[M-H]-	694.44533	1.21	['PE(13:0/20:5(5Z,8Z,11Z,14Z,17Z))', 'PE(15:1(9Z)/18:4(6Z,9Z,12Z,15Z))', 'PE(18:4(6Z,9Z,12Z,15Z)/15:1(9Z))', 'PE(20:5(5Z,8Z,11Z,14Z,17Z)/13:0)']
770.53492	2.10E+04	16.8	0.000	C42H78NO9P	[M-H]-	770.53415	1.01	['PS(O-16:0/20:3(8Z,11Z,14Z))', 'PS(O-18:0/18:3(6Z,9Z,12Z))', 'PS(O-18:0/18:3(9Z,12Z,15Z))', 'PS(P-16:0/20:2(11Z,14Z))', 'PS(P-18:0/18:2(9Z,12Z))']
341.19704	2.02E+04	16.3	0.001	C18H30O6	[M-H]-	341.19696	0.22	['2,3-Dinor-TXB2', '2,3-dinor, 6-keto-PGF1alpha']
910.61750	1.99E+04	16.0	0.000	C48H86NO9P	[M+Hac-H]-	910.61788	-0.41	['PS(P-20:0/22:4(7Z,10Z,13Z,16Z))']

794.49707	1.86E+04	15.0	0.000	C41H70NO8P	[M+Hac-H]-	794.49776	-0.87	['PE(14:0/22:6(4Z,7Z,10Z,13Z,16Z,19Z))', 'PE(16:1(9Z)/20:5(5Z,8Z,11Z,14Z,17Z))', 'PE(18:2(9Z,12Z)/18:4(6Z,9Z,12Z,15Z))', 'PE(18:3(6Z,9Z,12Z)/18:3(6Z,9Z,12Z))', 'PE(18:3(6Z,9Z,12Z)/18:3(9Z,12Z,15Z))', 'PE(18:3(9Z,12Z,15Z)/18:3(6Z,9Z,12Z))', 'PE(18:3(9Z,12Z,15Z)/18:3(9Z,12Z,15Z))', 'PE(18:4(6Z,9Z,12Z,15Z)/18:2(9Z,12Z))', 'PE(20:5(5Z,8Z,11Z,14Z,17Z)/16:1(9Z))', 'PE(22:6(4Z,7Z,10Z,13Z,16Z,19Z)/14:0)']

a Average intensity across all samples (n=8 control, n=6 low dose and n=8 high dose air-exposed samples).

b Fold change in intensity from control to *in vitro* air-exposed group.

c From t-test between control and air-exposed groups with a false discovery rate (FDR) of 5% to correct for multiple hypothesis testing.

d Calculated for the specified ion form of the empirical formula.

e Error between the observed and theoretical masses, presented as parts per million of the theoretical mass.
