# Supplementary material for: Defining the Baseline and Oxidant Perturbed Lipidomic Profiles of Daphnia magna
Source: Metabolites. 2017 Mar 15;7(1):11. doi: 10.3390/metabo7010011 (PMC5372214; doi:10.3390/metabo7010011)
Supplement: Supplementary file 1 [file metabolites-07-00011-s001.zip › Table S7.pdf]

Table S7. Summary of the top 20 (with the greatest fold change) putatively identified lipids of *D. magna* exposed to $CuSO_4$ *in vivo.*

Observed		Statistics		Annotation					
m/z	**Average intensity[a]**	**Fold change[b]**	**p-value[c]**	**Empirical formula**	**Ion form**	**Theoretical mass (Da)[d]**	**Mass error (ppm)[e]**	**Putative ID**	
443.31298	3.30E+04	8.1	0.001	C21H40N2O4	[M+Hac-H]-	443.312662	0.72	['N-palmitoyl glutamine']	
443.33810	1.96E+04	2.9	0.720	C23H44O4	[M+Hac-H]-	443.337814	0.65	['Tricosanedioic acid']	
476.27807	1.43E+05	2.0	0.166	C28H41NO4	[M+Na-2H]-	476.278228	-0.33	['15-HETE-DA', '15-HETE-VA']	
476.27807	1.43E+05	2.0	0.166	C23H44NO7P	[M-H]-	476.278266	-0.41	['LysoPE(18:2(9Z,12Z)/0:0)']	
512.29920	2.57E+04	1.9	0.817	C21H44NO7P	[M+Hac-H]-	512.299396	-0.38	['LysoPE(16:0/0:0)', 'PC(13:0/0:0)']	
492.36191	3.40E+04	1.9	0.720	C30H51NO2	[M+Cl]-	492.361381	1.08	['3-O-Aminopropyl-25-hydroxyvitamin D3']	
854.59165	3.88E+03	1.9	0.749	C45H82NO8P	[M+Hac-H]-	854.591661	-0.01	['PC(15:0/22:4(7Z,10Z,13Z,16Z))', 'PC(17:0/20:4(5Z,8Z,11Z,14Z))', 'PC(17:1(9Z)/20:3(8Z,11Z,14Z))', 'PC(17:2(9Z,12Z)/20:2(11Z,14Z))', 'PC(18:3(6Z,9Z,12Z)/19:1(9Z))', 'PC(18:3(9Z,12Z,15Z)/19:1(9Z))', 'PC(18:4(6Z,9Z,12Z,15Z)/19:0)', 'PC(19:0/18:4(6Z,9Z,12Z,15Z))', 'PC(19:1(9Z)/18:3(6Z,9Z,12Z))', 'PC(19:1(9Z)/18:3(9Z,12Z,15Z))', 'PC(20:2(11Z,14Z)/17:2(9Z,12Z))', 'PC(20:3(8Z,11Z,14Z)/17:1(9Z))', 'PC(20:4(5Z,8Z,11Z,14Z)/17:0)', 'PC(22:4(7Z,10Z,13Z,16Z)/15:0)', 'PE(18:0/22:4(7Z,10Z,13Z,16Z))', 'PE(18:2(9Z,12Z)/22:2(13Z,16Z))', 'PE(18:3(6Z,9Z,12Z)/22:1(11Z))', 'PE(18:3(9Z,12Z,15Z)/22:1(11Z))', 'PE(18:4(6Z,9Z,12Z,15Z)/22:0)', 'PE(20:0/20:4(5Z,8Z,11Z,14Z))', 'PE(20:1(11Z)/20:3(8Z,11Z,14Z))', 'PE(20:2(11Z,14Z)/20:2(11Z,14Z))', 'PE(20:3(8Z,11Z,14Z)/20:1(11Z))', 'PE(20:4(5Z,8Z,11Z,14Z)/20:0)', 'PE(22:0/18:4(6Z,9Z,12Z,15Z))', 'PE(22:1(11Z)/18:3(6Z,9Z,12Z))', 'PE(22:1(11Z)/18:3(9Z,12Z,15Z))', 'PE(22:2(13Z,16Z)/18:2(9Z,12Z))', 'PE(22:4(7Z,10Z,13Z,16Z)/18:0)']	
854.59165	3.88E+03	1.9	0.749	C47H86NO10P	[M-H]-	854.591661	-0.01	['PS(19:1(9Z)/22:2(13Z,16Z))', 'PS(20:3(8Z,11Z,14Z)/21:0)', 'PS(21:0/20:3(8Z,11Z,14Z))', 'PS(22:2(13Z,16Z)/19:1(9Z))']	
397.33270	2.40E+04	1.8	0.791	C24H46O4	[M-H]-	397.332334	0.92	['Axillarenic acid', 'Tetracosanedioic acid']	
397.33270	2.40E+04	1.8	0.791	C22H42O2	[M+Hac-H]-	397.332334	0.92	['(13Z)-Docosenoic acid', '22:1(7Z)', '22:1(9Z)', 'Cetoleic acid', 'trans-brassidic acid']	
474.26225	2.83E+05	1.6	0.014	C23H42NO7P	[M-H]-	474.262616	-0.77	['LysoPE(18:3(6Z,9Z,12Z)/0:0)', 'LysoPE(18:3(9Z,12Z,15Z)/0:0)']	
401.35515	1.70E+04	1.6	0.791	C25H50O	[M+Cl]-	401.355567	-1.04	['C25 6,7-Epoxy highly branched isoprenoid']	
817.55231	1.34E+03	1.6	0.652	C45H83O8P	[M+Cl]-	817.55196	0.43	['PA(20:1(11Z)/22:2(13Z,16Z))', 'PA(20:2(11Z,14Z)/22:1(11Z))', 'PA(20:3(8Z,11Z,14Z)/22:0)', 'PA(22:0/20:3(8Z,11Z,14Z))', 'PA(22:1(11Z)/20:2(11Z,14Z))', 'PA(22:2(13Z,16Z)/20:1(11Z))']	
956.73399	1.64E+03	1.6	0.768	C52H100NO8P	[M+Hac-H]-	956.732511	1.55	['PC(22:0/22:2(13Z,16Z))', 'PC(22:1(11Z)/22:1(11Z))', 'PC(22:1(13E)/22:1(13E))', 'PC(22:1(13Z)/22:1(13Z))', 'PC(22:2(13Z,16Z)/22:0)']	
447.32461	1.15E+04	1.6	0.835	C26H50O3	[M+K-2H]-	447.324602	0.02	['3-oxohexacosanoic acid']	
447.32461	1.15E+04	1.56	0.835	C29H46O2	[M+Na-2H]-	447.324449	0.36	['NA', '1alpha-hydroxy-24-methylvitamin D2 / 1alpha-hydroxy-24-methylergocalciferol', 'zymosterol intermediate 1c']	

895.59067	2.37E+03	1.5	0.802	C44H85O12P	[M+Hac-H]-	895.591722	-1.17	['PI(O-16:0/19:1(9Z))', 'PI(O-18:0/17:1(9Z))', 'PI(O-20:0/15:1(9Z))', 'PI(P-16:0/19:0)', 'PI(P-18:0/17:0)', 'PI(P-20:0/15:0)']
800.58035	1.34E+04	1.5	0.641	C42H80NO7P	[M+Hac-H]-	800.581096	-0.93	['PC(O-16:0/18:3(6Z,9Z,12Z))', 'PC(O-16:0/18:3(9Z,12Z,15Z))', 'PC(P-16:0/18:2(9Z,12Z))', 'PE(P-20:0/17:2(9Z,12Z))']
800.58035	1.34E+04	1.5	0.641	C44H84NO9P	[M-H]-	800.581096	-0.93	['PS(O-16:0/22:2(13Z,16Z))', 'PS(O-18:0/20:2(11Z,14Z))', 'PS(O-20:0/18:2(9Z,12Z))', 'PS(P-16:0/22:1(11Z))', 'PS(P-18:0/20:1(11Z))', 'PS(P-20:0/18:1(9Z))']
437.36363	3.29E+04	1.5	0.801	C25H46O2	[M+Hac-H]-	437.363634	-0.01	['22:2(5Z,9Z)(13Me,17Me,21Me)']
519.40523	3.25E+04	1.5	0.831	C30H52O3	[M+Hac-H]-	519.405499	-0.52	['(+)-24-Dammarene-3alpha,12beta,20S-triol', 'Myrrhanol A', 'Protopanaxadiol', 'Taraxastane-3beta,16beta,20beta-triol']
886.65415	4.08E+03	1.5	0.774	C47H90NO8P	[M+Hac-H]-	886.654261	-0.12	['PC(17:0/22:2(13Z,16Z))', 'PC(17:1(9Z)/22:1(11Z))', 'PC(17:2(9Z,12Z)/22:0)', 'PC(18:2(9Z,12Z)/21:0)', 'PC(19:0/20:2(11Z,14Z))', 'PC(19:1(9Z)/20:1(11Z))', 'PC(20:1(11Z)/19:1(9Z))', 'PC(20:2(11Z,14Z)/19:0)', 'PC(21:0/18:2(9Z,12Z))', 'PC(22:0/17:2(9Z,12Z))', 'PC(22:1(11Z)/17:1(9Z))', 'PC(22:2(13Z,16Z)/17:0)', 'PE(20:0/22:2(13Z,16Z))', 'PE(20:1(11Z)/22:1(11Z))', 'PE(20:2(11Z,14Z)/22:0)', 'PE(22:0/20:2(11Z,14Z))', 'PE(22:1(11Z)/20:1(11Z))', 'PE(22:2(13Z,16Z)/20:0)']
886.65415	4.08E+03	1.5	0.774	C49H94NO10P	[M-H]-	886.654261	-0.12	['PS(21:0/22:1(11Z))', 'PS(22:1(11Z)/21:0)']
458.34851	1.32E+05	1.5	0.720	C23H45NO4	[M+Hac-H]-	458.348713	-0.44	['L-Palmitoylcarnitine']
441.35845	3.79E+04	1.5	0.720	C24H46O3	[M+Hac-H]-	441.358549	-0.22	['Hydroxynervonic acid']

a Average intensity across all samples (n=8 control, n=6 low dose and n=8 high dose Cu exposed samples).

b Fold change in intensity from control to *in vitro* air-exposed group.

c From t-test between control and air-exposed groups with a false discovery rate (FDR) of 5% to correct for multiple hypothesis testing.

d Calculated for the specified ion form of the empirical formula.

e Error between the observed and theoretical masses, presented as parts per million of the theoretical mass.
