# Supplementary material for: Defining the Baseline and Oxidant Perturbed Lipidomic Profiles of Daphnia magna
Source: Metabolites. 2017 Mar 15;7(1):11. doi: 10.3390/metabo7010011 (PMC5372214; doi:10.3390/metabo7010011)
Supplement: Supplementary file 1 [file metabolites-07-00011-s001.zip › Table S1.pdf]

Supplementary Information

Internal mass calibration

The *m/z* list (Table S1) used to internally calibrate negative ion *D. magna* DIMS spectra during processing was created using fragmentation data. At low *m/z*, ions arising from the added ammonium acetate dominate with the deprotonated acetate dimer (*m/z* 119.03498) and the sodiated equivalent (*m/z* 141.01692). Free fatty acids are then seen at regular intervals from *m/z* 143.10775 to 283.26425. One lyso PE species (PE(18:3,0:0) was observed at *m/z* 474.26261. Unsaturated PE species were the most commonly observed phospholipid with seven species and five C^{13} isotopes yielding identifiable fragmentation patterns. Three PS and three PI species were also observed at higher *m/z*. Fragmentation patterns of many identified phospholipids showed isomeric chain chemistry. For example in the fragments of *m/z* 742.53923 carboxylate ions of fatty acids 18:0 (*m/z* 283), 18:1(*m/z* 281) and 18:2 (*m/z* 279) were all identified. Accurate mass matches for this peak included de-protonated PE(18:1(9Z),18:1(9Z)), PE(18:0,18:2(9Z,12Z)) and PE(18:2(9Z,12Z),18:0). These peaks all have equal mass with the only difference being double bond positions and are inseparable in DIMS. This isomeric situation can include many tens of species particularly with increasing double bond content. To avoid lengthy listing of species, bulk nomenclature will be used from here i.e. PE(18:1(9Z),18:1(9Z)), PE(18:0,18:2(9Z,12Z)) and PE(18:2(9Z,12Z),18:1) are combined to PE(36:2). All phospholipids manually identified had chain lengths of 34, 36 or 38 reflecting the prevalence of 16, 18 and 20 long fatty acids discussed in chapter 1 as combinations of these chain lengths can only yield 32, 34, 36, 38 and 40 carbon atoms (Bychek et al., 2005, Persson and Vrede, 2006). LipidBlast correctly annotated 8 of the 14 manually annotated phospholipids which were contained in the LipidBlast database with reverse dot product scores well in excess of the 600 required suggested by Kind et al. (2013).

Table S1 Identified lipid species used as internal calibrants for *D. magna* lipidome data processing. LipidBlast column shows the reverse dot product score indicating match success. NA indicates compound is not in the LipidBlast library, No ID indicates the compound is in the database but not identified

ID	Ion form	Theoretical *m/z*	LipidBlast rev dot score
Acetate dimer	$[M-H]^-$	119.03498	NA
Acetate dimer (C^{13})	$[M-H]^-$	120.03833	NA
Acetate dimer	$[M+Na-2H]^-$	141.01692	NA
8:0	$[M-H]^-$	143.10775	NA
10:0	$[M-H]^-$	171.13905	NA
12:0	$[M-H]^-$	199.17035	NA
16:0	$[M-H]^-$	255.23295	NA
16:0 (C^{13})	$[M-H]^-$	256.23630	NA
18:3	$[M-H]^-$	277.21730	NA
18:2	$[M-H]^-$	279.23295	NA
18:1	$[M-H]^-$	281.24860	NA
18:1 (C^{13})	$[M-H]^-$	282.25195	NA
18:0	$[M-H]^-$	283.62425	NA
18:0 (C^{13})	$[M-H]^-$	284.26760	NA
PE (18:3, 0:0)	$[M-H]^-$	474.26261	998
PE (34:3)	$[M-H]^-$	712.49232	961
PE (34:3) (C^{13})	$[M-H]^-$	713.49567	NA
PE (34:2)	$[M-H]^-$	714.50797	984
PE (36:6)	$[M-H]^-$	734.47663	No ID
PE (36:6) (C^{13})	$[M-H]^-$	735.47998	NA
PE (36:5)	$[M-H]^-$	736.49228	No ID
PE (36:5) (C^{13})	$[M-H]^-$	737.49563	NA
PE (36:4)	$[M-H]^-$	738.50793	983
PE (36:4) (C^{13})	$[M-H]^-$	739.51128	NA
PE (36:3)	$[M-H]^-$	740.52358	No ID
PE (36:3) (C^{13})	$[M-H]^-$	741.52693	NA
PE (36:2)	$[M-H]^-$	742.53923	994

PS (36:3)	$[M-H]^-$	784.51341	No ID
PS (36:2)	$[M-H]^-$	786.52906	No ID
PS (36:0)	$[M-H]^-$	790.56036	985
PI (36:4)	$[M-H]^-$	857.51855	No ID
PI (36:3)	$[M-H]^-$	859.53420	839
PI (38:5)	$[M-H]^-$	883.53420	807

Peak annotation of *D. magna* baseline lipidome

Isomeric overlap is rife within lipid species: PC/PE, PS/PC, and PA/PG all have isomeric overlap. It has been well reported that PC species do not form de-protonated ions with ESI (Pulfer and Murphy, 2003) and so any [PC-H]- annotations were also removed from the baseline annotation, there were an abundance of these as [PC-H]- isomerically overlaps with [PE-H]- which should be highly expressed (Bychek et al., 2005). Fragmentation data added a degree of confidence to the putative annotation of 63 of 148 PC/PS species (Table S4). There was almost total overlap between [PGH]– and [PA+Ac]–, however, PA adducts were almost exclusively odd chained 2 of 17 and de-protonated PGs almost exclusively even chained 15 of 16 suggesting that the latter are correct and the PA adducts false positives as odd fatty acyl chains are rare in *D. magna*. Finally, there is isomeric overlap between plasmalogens with a vinyl ether bond at the sn1 position and the lipid subspecies with an ether bond at sn1 should it contain an extra double bond on one of the chains (e.g. PE(P-34:3) & PE(O-34:4)). These species differ only by a single double bond position and as such are extremely challenging to separate and so are treated as single entities in this instance. Chain length and double bond number both show identifiable but different trends across phospholipid classes. Chain length parity for [PA+Ac]- annotations is predominantly odd (87 % odd), however, this was the only lipid class where that was the case with parity predominantly even in all other classes [PC+Ac]- (53 % even), [PE-H]- (71 % even), [PGH]- (93 % even), [PI-H]- (71% even) and [PS-H]- (55 % even). A low ratio of odd to even chain lengths is expected given the vast prevalence of even chained fatty acids in *D. magna* with only 15:0 and 17:0 being reported previously in relatively small concentrations (Bychek and Gushchina, 1999, Persson and Vrede, 2006). [PA+Ac] – ion forms were retained in baseline annotation, however, the isomeric overlap with [PG-H]- and extremely high odd chain abundance indicates these annotations may be false positives as mentioned above. The relatively equal ratios in PC and PS are probably related to the presence of even chained [PE+Ac]- which would appear at the same *m/z* values as odd [PC+Ac]- and [PS-H]-. However, in the cases where odd chains were annotated, the adjacent even chains consistently dominated in terms of intensity, as expected, in all classes but PA.

References

Bychek E. A., Gushchina, I. A., 1999. Age-dependent changes of lipid composition in *Daphnia magna*. *Biochem. Mosc.* 64**,** 543-545

Bychek E. A., Dobson G. A., Harwood J. L., Guschina I. A., 2005. *Daphnia magna* can tolerate short-term starvation without major changes in lipid metabolism. *Lipids* 40**,** 599-608

Kind T., Liu K.-H., Lee D. Y., Defelice B., Meissen J. K., Fiehn, O., 2013. LipidBlast in silico tandem mass spectrometry database for lipid identification. *Nat. Methods,* 10(8), 755-758

Persson J., Vrede, T., 2006. Polyunsaturated fatty acids in zooplankton: variation due to taxonomy and trophic position. *Freshw. Biol.* 51**,** 887-900

Pulfer M., Murphy R. C., 2003. Electrospray mass spectrometry of phospholipids. *Mass Spectrom. Rev.* 22**,** 332-364
